# Residential fungal β-(1,3)-D-glucan exposure is associated with decreased pulmonary function in fibrotic pulmonary sarcoidosis

**DOI:** 10.21203/rs.3.rs-5220174/v1

**Published:** 2024-10-22

**Authors:** Emma M Stapleton, Nervana Metwali, Michael Shlossman, Linder Wendt, Alejandro A Pezzulo, Nabeel Y Hamzeh, Alejandro P Comellas, Peter S Thorne, Alicia K Gerke

**Affiliations:** University of Iowa; University of Iowa; University of Iowa; University of Iowa; University of Iowa; University of Iowa; University of Iowa; University of Iowa; University of Iowa

**Keywords:** Sarcoidosis, pulmonary fibrosis, indoor air, bioaerosol, β-(1,3)-D-glucan

## Abstract

**Background:**

Sarcoidosis is a multi-system disease frequently affecting the lungs. It is thought to be mediated by gene-environment interaction; for example, epidemiological data show organic aerosol exposure increases risk of pulmonary sarcoidosis.

**Research Question:**

Does exposure to bioaerosol associate with worse lung disease in patients with pulmonary sarcoidosis?

**Research Question:**

Using an observational, cohort study design, we measured residential exposure to fungal and bacterial cell wall material, β-(1,3)-D-glucan (BDG) and endotoxin, respectively, in healthy control subjects and those with pulmonary sarcoidosis. In the case cohort, we compared bioaerosol concentrations to pulmonary disease severity, assessed by pulmonary function testing, qualitative chest computed tomography (CT), and serum biomarkers. Log-transformed bioaerosol concentrations were compared to lung function and significance and correlation determined by Pearson correlation.

**Results:**

Homes of subjects with sarcoidosis had higher BDG and endotoxin concentrations than control subjects. Patients with significant pulmonary fibrosis had greater disease severity (Wasfi severity score, visual analogue scale) and reduced pulmonary function compared to those without fibrosis (all *P*<0.01). Residential fungal BDG correlated with declining FVC, only in patients with fibrosis on CT imaging (*P*=0.02). Survey data revealed higher BDG concentrations were found in homes of cat-owners, and the number of houseplants owned correlated with declines in FVC and FEV_1_ (*P*=0.05 and 0.02, respectively). In patients without fibrosis, eight inflammatory markers correlated with BDG (6CKine/CCL21, IL-9, IL-17F, IL-21, IL-28A, I-309, MIP-1β, TARC), while in those with pulmonary fibrosis, BDG correlated with two inflammatory markers (Eotaxin-3, M-CSF), suggesting immune anergy to inhaled antigens in patients with fibrosis.

**Interpretation:**

In patients with pulmonary fibrosis, disease severity was correlated with residential exposure to fungal cell wall material, but not gram-negative bacterial cell wall material. These patients may experience immune anergy to inhaled antigens.

## INTRODUCTION

Sarcoidosis is a multi-system inflammatory granulomatous syndrome principally affecting the lungs; the disease has no available cure [1]. It’s exact cause remains unknown, but various triggers of sarcoidosis-like granulomatous response have been proposed, including environmental antigens (e.g., silica, talc, mycobacterial [2,3], and *Cutibacterium* (previously *Propionibacterium)* acnes [4]. Gene-environment interaction is strongly suggested [5], with exposures ranging from inorganic [6], to organic antigens [4,7]. Several studies, including the multi-center epidemiological study—A Case Control Etiologic Study of Sarcoidosis (ACCESS)— associate exposure to high humidity, water damage and mold/musty odor with sarcoidosis and pulmonary-specific sarcoidosis [5,8–11]. Additionally, previous work found patients with newly diagnosed and active disease had greater residential microorganism biomarker concentrations (non-specific N-acetylhexosaminidase) than controls and those without disease recurrence [12]. Lastly, bronchoalveolar lavage (BAL) from patients with Löfgren’s syndrome was found to have a T-cell receptor epitope recognized and stimulated by an *Aspergillus nidulans*-specific peptide [13].

Therefore, a plausible source of perpetuating antigens are bioaerosols – airborne particulate matter derived from microbes, plants or animals, including inhalant allergens, pollens, fungal spores, bacteria, and viruses [14]. Fungal-derived bioaerosols can vary widely in spore size, culturability, phyla, and branching pattern, but purified polysaccharide fungal cell wall material (β-d-glucan, BDG) is a common fungal biomarker capable of activating the immune system via pattern recognition receptors. Similarly, exposure to pro-inflammatory gram-negative bacterial endotoxin can be estimated by quantifying lipopolysaccharide cell wall material [15].

Despite strong epidemiological data, research efforts to systematically assess personal exposure to proposed aerosolized antigens are lacking [11]. **Research Question:** Does exposure to bioaerosol associate with worse lung disease in patients with pulmonary sarcoidosis? **Hypothesis:** Exposure to residential bioaerosols differentially affects disease course in subjects with pulmonary fibrosis compared to those without fibrosis. To test this hypothesis, we measured fungal and bacterial bioaerosols in residences of control subjects and those with pulmonary sarcoidosis and compared BDG and endotoxin concentrations to pulmonary function outcomes and serum biomarkers.

## MATERIALS AND METHODS

### Cohort.

In our observational cohort study, adult patients over the age of 18 with biopsy-proven pulmonary sarcoidosis were enrolled at the University of Iowa Interstitial Lung Disease clinic. This study has obtained ethics approval from the IRB review board-01 review board #IRB00000099. Study protocol followed the University of Iowa’s Institutional Review Board requirements (IRBs: #201709743, 201909726, 202009330, Approved by IRB Chair). Subjects completed informed written consent prior to study participation for all study procedures. Homes were located in Iowa and surrounding states (e.g., Illinois, Wisconsin). Control subject homes were from a similar geographical area. Subjects with sarcoidosis completed a survey interrogating daily habits and exposure sources (N=55, survey included in **Appendix A, Supplemental Material**).

### Pulmonary Function Tests (PFTs).

PFT measurements (pre-bronchodilator percent predicted forced vital capacity, FVC, forced expiratory volume in one second FEV_1_, FVC/FEV_1_ ratio, and diffusing capacity of carbon monoxide, DLCO) temporally nearest bioaerosol measurement were extracted from the medical records.

### Disease severity assessment and quantification.

Patient medical records were reviewed to extract demographics and clinical indicators of disease severity, including details of confirmed or probable extrapulmonary organ involvement, disease duration (according to diagnostic guidelines [16]), and the use of immunosuppression (IS). This information was used to calculate a Wasfi Severity Score according to the multivariate model derived by Wafsi et al. [17] (for details, see **Appendix A, Supplemental Methods**). The presence or absence of lung fibrosis was qualitatively assessed by a clinical radiologist using clinical chest CT scans and confirmed by expert sarcoidosis physician (AKG).

Additionally global disease severity was assessed using a tool applied in various diseases [17,18]. Patients were rated for disease severity by visual analog scale (VAS). An expert clinician (AKG) blinded to exposure data marked a 5-inch horizontal line, whereby 0 indicated incidentally discovered asymptomatic disease and 10 denoted severe end-stage organ dysfunction (e.g., progressive fibrotic lung disease requiring consideration for transplant). The distance from 0 to the mark (in inches), multiplied by 2 defined a patient’s VAS score. We included sarcoidosis health questionnaire (SHQ) data regarding daily, emotional and physical function [19] in those who completed these clinical assessments.

### Residential bioaerosol exposure assessment.

Electrostatic dust collectors (EDCs) were placed at approximate breathing zone height using sterile endotoxin-free gloves in the primary household occupied room for 2 weeks [20], and subsequently mailed back to our University. EDC cloths were then removed and stored at −20°C. Field and lab blanks (7% of samples) were collected and analyzed identically to the samples.

### EDC β-(1,3)-d-glucan extraction.

β-(1,3)-d-glucan (BDG) was determined using the Glucatell^®^ assay (Associates of Cape Cod, Inc., East Falmouth, MA, USA) of air samples collected on the EDC cloths, based on a modification of the Limulus Amebocyte Lysate factor G pathway. Room temperature samples were eluted into LAL reagent water (Lonza RTP, Durham, NC, USA), with vortexing and shaking on an orbital shaker (30 min.) followed by sonication (30 min., 22°C), and further shaking (10 min.). Extracts were then centrifuged (15 min., 600 × g, 4°C) and mixed with NaOH (final 0.3N), then shaken (4°C, 30 min.), centrifuged (15 min., 600 × g), and analyzed. BDG was quantified at 37°C using a multimode microplate reader (Molecular Devices M5) at 550 nm. The reagent does not react to other polysaccharides, including β glucans with different glycosidic linkages and is a well-established β-(1,3)-d-glucan measurement method [21].

### EDC endotoxin extraction.

Endotoxin was extracted from EDCs by established kinetic chromogenic Limulus amebocyte lysate assay methodology [22,23], with a minor modification (additional 30 min. sonication of suspended cloths).

### Phlebotomy and serum cytokine processing.

Venipuncture was performed on consented individuals within approximately one year of residential measurement. Of our case cohort, serum was obtained from 36 individuals (no pulmonary fibrosis, N=22; with pulmonary fibrosis N=14). Serum and plasma separator tubes were centrifuged (10 min., 2000 RPM), deidentified and stored at −80°C. Serum was centrifuged (10 min., 1000g, 4°C) then assayed using the Human Cytokine/Chemokine 71-Plex Discovery Assay^®^ Array and the Human Supplemental Biomarker 10-Plex Discovery Assay^®^ Array (Eve Technologies, Alberta, CA, for details, see **Appendix A, Supplemental Methods**).

### Statistical analyses.

Population size was determined by power analysis (G*Power version 3.1.9.7) based on a publication of a Slovenian population with sarcoidosis – the study measure units of non-specific NAHA from 4hr. filter measurements [12]. Control subject homes had a mean NAHA concentration of 10 U/m^3^ (N=30, SD=6) and homes of subjects with newly diagnosed sarcoidosis (N=55, SD=37) was 33.6 U/m^3^. Our two-tailed *a priori t*-test analysis included α=0.05 and a power of 0.8. We allocated 2 patients:1 control subject, for a total sample size of 48 (32 with sarcoidosis, 16 without). To assure sufficient power, we recruited 84 subjects (59 with sarcoidosis, 25 without).

Residential characteristics were assessed using R (v.4.3.3) by comparing raw BDG values across various strata via Kruskal-Wallis rank sum tests and Wilcoxon rank sum tests. Comparisons between subjects with sarcoidosis and control subjects were made using Fisher’s exact test for categorical variables and using Welch two sample *T*-tests for continuous variables. Right-skewed data were log-transformed before *T*-tests were implemented to properly meet *T*-test assumptions. The same tests were used to compare characteristics of non-fibrotic and fibrotic patients. Remaining statistical analyses were performed using GraphPad Prism (v.10.2.3). Log-transformed bioaerosol concentrations (BDG, endotoxin) were compared to lung function and significance and correlation determined by Pearson correlation. Trend lines were determined by simple linear regression. Differences in residential bioaerosol levels between those with and without significant pulmonary fibrosis were analyzed by unpaired *T*-tests. One-way ANOVA with Tukey’s adjustment for multiple comparisons was used to assess the effect of season on bioaerosol exposures. Next, BDG and endotoxin concentrations were log transformed and compared to lung function and log-transformed houseplant number +0.1 by simple linear regression. Serum cytokine data were checked for outliers by ROUT, and definitive outliers removed (Q=0.1%), then their association with log-transformed BDG concentrations was evaluated by Pearson r. Herein we present data with a *P* value ≤0.11 and considered significance at *P*≤0.05.

## RESULTS

### Demographics and sarcoidosis disease severity.

Year-round bioaerosol sampling occurred between March 2021-May 2024. Measurements from 59 homes of subjects with pulmonary sarcoidosis and 25 without sarcoidosis were included. The population with sarcoidosis was older (56.7 vs. 42.2 years, *P*<0.01) compared to that without sarcoidosis. Homes of subjects with sarcoidosis had significantly greater BDG and endotoxin concentrations than control subject homes (BDG geometric mean: 1,320.0 vs. 160.5 ng/m^2^, *P*<0.01; endotoxin geometric mean: 934.8 vs. 263.0 EU/m^2^, *P*<0.01; **Table 1**). Lab and field blank concentrations were well below sample values (BDG geometric mean=0.03 ng/mL, endotoxin geometric mean= 0.31 EU/mL).

We next evaluated differences in our case cohort between those with sarcoidosis experiencing significant fibrotic pulmonary disease compared to those without pulmonary fibrosis. Subjects with fibrotic pulmonary disease were predominantly male, and disease severity was significantly greater, reflected by higher VAS and Wasfi score (*P*<0.01), and lower lung function (*P*<0.01, all). More males were on IS (60% vs. 41% of cohort, data not shown). There were no significant differences in bioaerosol concentrations between the two groups (**Table 2**). Subjects with pulmonary fibrosis also tended toward less extrapulmonary organ involvement.

### Behavioral and housing characteristics associated with BDG concentrations.

To assess bioaerosol sources, residential BDG concentrations were compared to housing and behavioral characteristics in our case cohort (**Appendix A, Supplemental Material**). Residential BDG concentrations were positively associated with cat ownership and daily cooking, *P*<0.05. Number of days anything is burned in the house tended to positively associate with BDG concentration (*P*=0.09), while use of plastic cooking materials (e.g., non-stick), ionizing air purifiers, and recent construction trended as protective (*P*=0.08, *P*=0.11, *P*=0.09, respectively; **Table 3**).

### Association between lung function and bioaerosol exposure.

**Figure 1:**
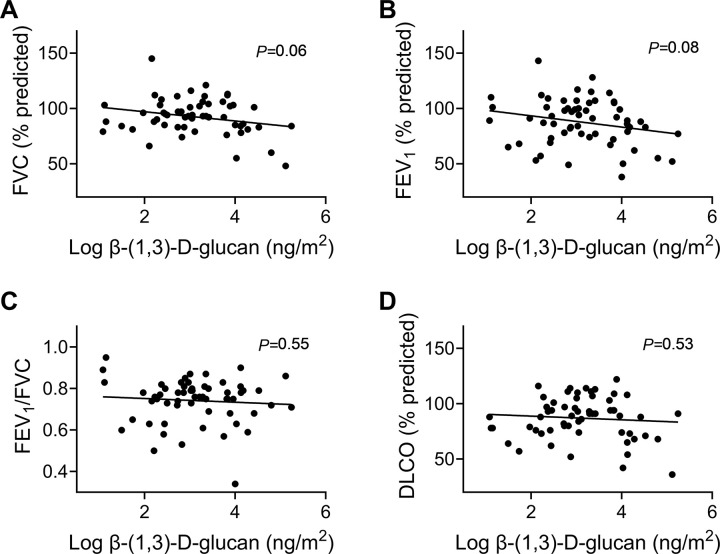
Residential β-(1,3)-d-glucan exposure and lung function in cohort of participants with pulmonary sarcoidosis. **A)** Percent predicted FVC tended to decrease as BDG concentration increased, *P*=0.06; **B)** Percent predicted FEV_1_ tended to decrease as residential BDG concentration increased, *P*=0.08; **C)** Percent predicted FEV_1_/FVC was not related to residential BDG concentrations; *P*=0.55; **D)** FEV_1_/FVC was not related to log-transformed BDG concentrations, *P*=0.53; N=59.

Next, residential exposure to BDG and endotoxin was compared to lung function in our case cohort. Overall, log-transformed BDG exposure tended to correlate weakly with declines in percent predicted FVC and FEV_1_ across the cohort, (*P*=0.06 and 0.08, respectively; Fig. 1 A–B). Lung function did not correlate with endotoxin concentrations (**Fig. S1**).

### Relationship between residential bioaerosol and sarcoidosis pulmonary severity.

**Figure 2:**
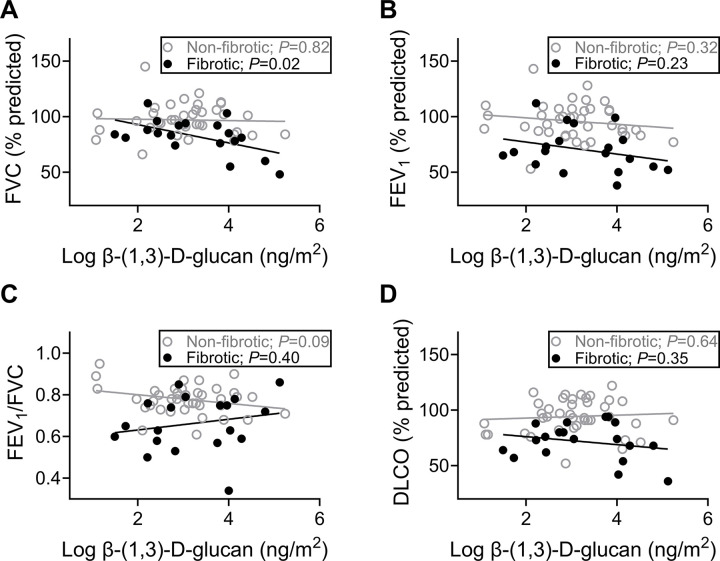
In participants with fibrotic pulmonary sarcoidosis, increased residential exposure to β-(1,3)-d-glucan is correlated with lower FVC. **A)** Percent predicted FVC did not decrease in relation to residential BDG in participants without fibrosis, but did in those with fibrosis (*P*=0.82 and *P*=0.02); **B)** Percent predicted FEV_1_ was not related to log-transformed BDG concentrations in either group (non-fibrotic, *P*=0.32; fibrotic, *P*=0.23); **C)**FEV_1_/FVC tended to be related to residential BDG concentrations in those without fibrosis but was unrelated in those with fibrosis (non-fibrotic, *P*=0.09; fibrotic, *P*=0.40); **D)** Percent predicted DLCO was not related to log-transformed BDG concentrations in either group (non-fibrotic, *P*=0.64; fibrotic, *P*=0.35); non-fibrotic N=40, fibrotic N=19.

We next asked whether residential bioaerosol correlated with lung function depending on pulmonary phenotype. A decline in percent predicted FVC significantly correlated with residential BDG concentration only in those with pulmonary fibrosis (*P*=0.02; Fig. 2A). FEV_1_/FVC tended to decline in correlation with residential BDG concentration in the non-fibrotic group (*P*=0.09; Fig. 2C). No correlations with endotoxin were observed irrespective of pulmonary fibrosis status (**Fig. S2**).

**Figure 3:**
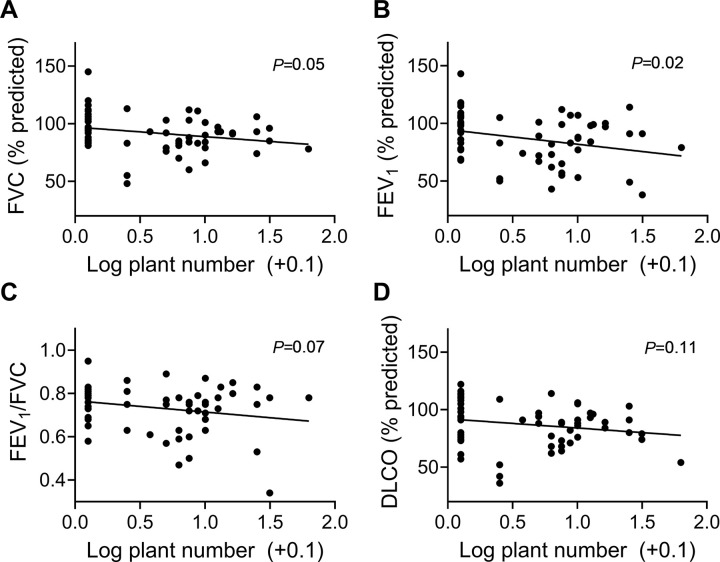
Lung function decline was associated with number of houseplants owned. Houseplant ownership significantly correlated with pulmonary fibrosis. Participant-reported number of houseplants owned were log transformed and compared by simple linear regression to **A)** Percent predicted FVC, *P*=0.05; **B)** Percent predicted FEV_1_, *P*=0.02; **C)** FEV_1_/FVC, *P*=0.07, and **D)** Percent predicted DLCO, *P*=0.11.

Because houseplants can be reservoirs for bioaerosol [24], we next tested whether there was a linear relationship with lung function and the number of houseplants owned, which was not normally distributed. Number of houseplants was associated with a decline in FVC and FEV_1_ (*P*≤0.05) and a trend for FEV_1_/FVC (*P*=0.07) (Fig. 3). Furthermore, houseplant ownership was observed more frequently in homes of subjects with pulmonary fibrosis (86% vs. 53%, *P*=0.01), data not shown.

### Correlation of biomarkers with residential exposures.

Within our entire case cohort, nine cytokines and chemokines positively correlated with residential BDG exposure (CCL21, Eotaxin-3, G-CSF, M-CSF, IL-9, IL-15, IL-21, IL-28A, FLT-3L), while two negatively correlated (I-309 and TARC). In non-fibrotic disease, five cytokines positively correlated with residential BDG exposure (CCL21, IL-9, IL-21, IL-17F, IL-28A) and three negatively correlated (I-309, MIP-1β and TARC). In subjects with pulmonary fibrosis, only two cytokines positively correlated with residential exposures – Eotaxin-3 and M-CSF (**Table 4**).

Of subjects with sarcoidosis in whom blood was drawn and residential bioaerosol measured, 19% without fibrosis were prescribed non-steroidal IS, 8% were prescribed steroidal IS and 8% were prescribed both at the time of blood draw. Fifty-eight percent were not prescribed either class of medication. Of subjects with fibrosis, 35% were prescribed non-steroidal IS, 12% prescribed steroidal IS and 12% were prescribed both. Forty-one percent were not taking either class of medication (data not shown).

## DISCUSSION

Sarcoidosis pathogenesis has long been thought to be influenced by inhaled environmental antigens, yet direct measurement of residential exposures is lacking. Our study revealed homes of participants with pulmonary sarcoidosis had significantly greater bioaerosol concentrations than those without sarcoidosis. Among our case cohort, pulmonary disease severity was associated with residential fungal BDG concentration. Specifically, residential BDG associated with FVC decline in subjects with pulmonary fibrosis, but not in those without fibrosis. This relationship was not observed with gram-negative bacterial endotoxin concentration. Subjects with sarcoidosis-related pulmonary fibrosis seem to be more susceptible to residential bioaerosol exposure compared to those with a milder or self-resolving phenotype. These data suggest fungal antigens as potential agents responsible for perpetuating sarcoidosis pulmonary disease severity.

Increasing evidence points towards a role for fungal antigens to incite or perpetuate pulmonary disease. For example, the specific glucan analyzed in our study (β-(1,3)-d-glucan) can increase inflammatory infiltrate into the airway, and prime cells for enhanced inflammatory response to secondary stimuli in a murine model [25]. Bioaerosol has also been shown to cause and worsen pulmonary fibrosis [26,27] and in patients with sarcoidosis, peripheral blood mononuclear cell (PBMC) production of IL-12 is increased after stimulation with particulate-BDG [28]. In our study, eight cytokines (CCL21, a T-cell chemoattractant, IL-9, IL-17F, IL-21, IL-28A, I-309, MIP-1β, and TARC) significantly correlated with BDG exposure in those without fibrosis, while only two cytokines (eotaxin-3 and M-CSF) were correlated in those with fibrosis (**Table 4**). This muted inflammatory response to inhaled antigens in subjects with pulmonary fibrosis might indicate impaired immune surveillance toward exogenous ligands. Impaired regulatory T-cell anergy has been previously reported in sarcoidosis [29].

Across the case cohort, serum IL-28A concentrations consistently correlated with residential BDG exposures (**Table 4**). IL-28A is structurally similar to IL-10 family cytokines, binding with the IL-10 receptor β chain. It is considered a Type III interferon (IFN-λ2) with potent anti-viral and antibacterial activity [30]. Type-III interferons protect epithelial barriers, which first encounter incoming pathogens including aerosolized BDGs [31]. They can promote Th1 skewing [32], a well-established pathway for sarcoidosis pathogenesis [33]. Because IL-28A can be activated by bacterial components, and increases TLR2 expression [34], BDG binding to pattern recognition receptors likely mediate the increased IL-28A concentrations observed with BDG exposure.

Interestingly, Eotaxin-3, a chemokine for eosinophils, consistently correlated with residential BDG exposure across the case cohort, despite previous work showing increased IFN-λ reduces Th2 cytokines. Eotaxin-3 is characteristic of allergic response and is a key chemokine in the fibrotic process; for example, airway eotaxin-3 promotes fibroblast migration [35,36]. Of note, fungal *Aspergillus fumigatus* allergen injection is a murine model to study fibrosis of the esophagus, which eotaxin-3 contributes to [36,37]. Accordingly, eotaxin-3 response to inhaled fungal allergen might contribute to worsening pathology in sarcoidosis and warrants further study.

In fibrotic disease, macrophage colony-stimulating factor (M-CSF) was the most significantly associated with BDG exposure. M-CSF is thought to regulate tissue macrophage and monocytes [38] and can lead to less differentiated monocytes (compared to GM-CSF) [39]. It has been reported patients with sarcoidosis have more intermediate monocytes, which are high in MHC-II expression, responsible for binding and presenting antigens to CD4+ T cells [40]. MHC-II alleles have previously been shown to interact with mold/musty odor exposure and pulmonary sarcoidosis risk [5].

Several sources of bioaerosol and environmental factors affecting disease severity were gleaned by survey assessment. Exposure to bioaerosol frequently increased with activities known to generate combustion aerosol. For example, burning of candles/other tended to correlate with increased BDG concentrations, and daily cooking correlated with increased BDG concentrations (**Table 3**). Cooking leads to combustion aerosol exposures [41], which can adsorb bioaerosol [42]. Cat owners had higher BDG concentrations, likely related to bioaerosol contained in animal hair and skin [43,44]. Survey data also demonstrated a higher proportion of subjects with pulmonary fibrosis owned houseplants, which can be mold reservoirs [24]. The number of plants participants owned was significantly correlated with declined FEV_1_ and trends for FVC, FEV_1_/FVC (Fig. 3). These associations may warrant further exploration in future exposure assessment work.

**Study strengths.** Ours is the first to directly measure specific bacterial and mold residential bioaerosol in sarcoidosis; our study revealed a novel relationship between residential BDG and sarcoidosis pulmonary severity. **Study limitations.** Participants were responsible for EDC deployment, and although we contacted participants to confirm adherence, we cannot exclude the possibility that some participants incorrectly followed protocol instructions. Second, sampling occurred throughout the year, which could skew results [45]. However, bioaerosol concentrations did not vary based on sampling season (*p*>0.91), and within-home temporal variability can be minimal [46]. Third, although we have strong association with lung function and houseplant number/other home features, this does not confirm causality. Fourth, our study solely measured bioaerosol. Metals, such as aluminum have been implicated in sarcoidosis [47]. Homes in the same geographical area have previously been characterized and aluminum was the most highly enriched metal [48]. Interaction between metal and mold exposure in homes should be considered in future work. Fifth, ionizing air purifiers, occasionally used in this study, can generate ozone – a potential confounder for PFT results. Conversely, ionizers effectively reduce residential combustion aerosol [49], and their use tended to correlate with a 10-fold reduction in BDG concentrations. Sixth, biomarker collection did not always occur simultaneously with residential measurements, and patients were on various therapies. To overcome these weaknesses, we excluded cytokine outliers and values below the assay’s limit of detection and removed definitive outliers (ROUT, Q=0.1%) and compared these to log-transformed bioaerosol concentrations. Further, rates of IS use were similar between phenotypes. Exclusion criteria may have resulted in underestimation of results, e.g., extremely high, or low concentrations of specific biomarkers being overlooked, yet they provide more confidence in accuracy of findings. Last, our findings are from a relatively small heterogenous cohort; however, power was determined a priori, and we oversampled to accommodate residential, demographic, and phenotypic variability. Future work should include a more diverse cohort to confirm findings.

## CONCLUSION

Bioaerosol concentrations were greater in homes of subjects with sarcoidosis than control subjects. In our case cohort, lung function of subjects with fibrotic pulmonary disease associated with residential fungal BDG. Th1 and Th2 cytokines correlated with BDG exposure, but far fewer inflammatory molecules correlated with exposure in those with pulmonary fibrosis, potentially suggestive of T-cell exhaustion. Further investigation of bioaerosol sources is warranted, as interventions can be designed and tested to see if disease course is modifiable by environmental remediation.

## Data Availability

Datasets and materials are available and can be accessed by contacting co-corresponding author at emma-stapleton@uiowa.edu.

## ABBREVIATIONS

BDG: β-d-glucan
DLCO: Diffusing capacity of carbon monoxide
FEV_1_: Forced expiratory volume in one second
FEV_1_/FVC: The ratio of FEV_1_ over forced vital capacity
FVC: Forced vital capacity
IL: Interleukin
MHC-II: Major Histocompatibility Complex
PBMC: Peripheral blood mononuclear cell
SHQ: Sarcoidosis Health Questionnaire
Wasfi Severity Score: A previously established method of evaluating sarcoidosis disease severity
VAS: Visual Analogue Scale
